# Torsade de Pointes: How Much Do Psychotropics Really Contribute?

**DOI:** 10.1192/j.eurpsy.2025.413

**Published:** 2025-08-26

**Authors:** J. Brouillette, S. Cyr, C. Fiset, R. Tadros, L. Abdelaziz, A. Minichiello, M. Bouvier, S. Levesque, C. Daval, V. Lavigne, P. Khairy, S. Nattel

**Affiliations:** 1 Montreal Heart Institute, Montreal, Canada

## Abstract

**Introduction:**

Based on potential risk of torsade de pointes (TdeP; a rare arrhythmia), regulatory agencies have issue warning for two specific antidepressants. This has translated in clinic as a class effect in that every antidepressant monography or guideline warns against this side effect. Current data suggest that excessive caution in the face of this undesirable effect could have a deleterious effect on mortality. Most research on antidepressant/antipsychotic drugs and TdeP is based on its intermediate marker, the corrected QT interval (QTc) on the electrocardiogram, or case reports.

**Objectives:**

Our objective is to measure the contribution of psychotropic drugs (antidepressants and antipsychotics) to the arrhythmia itself, and measure its weight among all other risk factors.

**Methods:**

We completed a retrospective case-control study at the Montreal Heart Institute, with a 1:3 ratio (n=440). We performed hierarchical logistic regression for TdeP (cases vs controls), and included the following independent variables: sex, age, hypokalemia, acute myocardial infarction, left ventricular dysfunction, hepatic failure and/or renal failure, other QTc-prolonging drugs, and psychotropic drugs. We then calculated population attributable risks (PAR).

**Results:**

In our final model which adjust risk factors for one another, women, acute myocardial infarction, hypokalemia, left ventricular dysfunction, QTc-prolonging drugs, and use of ≥2 antidepressants were significantly associated with TdeP. Age, hepatic and/or renal failure and antidepressants/antipsychotic drug monotherapy were not. The PAR for use of ≥2 antidepressants was 6.3%, while those of other QTc-prolonging drugs, female sex, and left ventricular dysfunction were 55.1%, 41.8%, and 25.6%, respectively.

**Conclusions:**

When adjusting for concomitant risk factors, monotherapy with antidepressant or antipsychotic drugs is not associated with TdeP. On its side, the use of ≥2 antidepressants is associated with TdeP, with a PAR of lesser magnitude than sex, left ventricular dysfunction, and other QTc-prolonging drugs. This research provides a more nuanced perspective on the relationship between psychotropic drugs and the occurrence of this arrhythmia.

**Disclosure of Interest:**

None Declared

